# Deep Neuromuscular Block Improves Surgical Conditions during Bariatric Surgery and Reduces Postoperative Pain: A Randomized Double Blind Controlled Trial

**DOI:** 10.1371/journal.pone.0167907

**Published:** 2016-12-09

**Authors:** Bart Torensma, Chris H. Martini, Martijn Boon, Erik Olofsen, Bas in ‘t Veld, Ronald S. L. Liem, Mireille T. T. Knook, Dingeman J. Swank, Albert Dahan

**Affiliations:** 1 Department of Anesthesiology, Leiden University Medical Center, Leiden, The Netherlands; 2 Department of Surgery, Dutch Obesity Clinic West, The Hague, The Netherlands; 3 Department of Anesthesiology, Haaglanden Medical Center, The Hague, The Netherlands; Cardiff University, UNITED KINGDOM

## Abstract

**Background:**

It remains unknown whether the administration of a deep neuromuscular block (NMB) during bariatric surgery improves surgical conditions and patient outcome. The authors studied the effect of deep *versus* moderate NMB in laparoscopic bariatric surgery on surgical conditions and postoperative pain.

**Methods and Results:**

One hundred patients scheduled to undergo elective bariatric surgery were randomized to a deep NMB (post-tetanic-count 2–3) or a moderate NMB (train-of-four 1–2). The quality of the surgical field was scored using the Leiden-Surgical Rating Scale (L-SRS), a 5-point scale ranging from 1 (extremely poor conditions) to 5 (optimal conditions). Three surgeons scored the L-SRS at 10-min intervals during surgery; postoperative pain scores were obtained in the postanesthesia-care-unit (PACU) and on the ward. Mean (95% confidence interval) L-SRS scores in moderate NMB 4.2 (4.0–4.4) *versus* 4.8 (4.7–4.9) in deep NMB (p < 0.001). Moderate NMB resulted in 17% of scores at L-SRS scores of 1–3, while deep NMB resulted in 100% scores at the high end of the L-SRS (4–5). Deep NMB led to improved pain scores in the PACU (4.6 (4.2–4.9) *versus* 3.9 (3.6–4.4), p = 0.03) and reduced shoulder pain on the ward (1.8 (1.5–2.1) *versus* 1.3 (1.1–1.5), p = 0.03). A composite score of pain and opioid use in the PACU favoured deep NMB (p = 0.001).

**Conclusions:**

In bariatric surgery, deep relaxation has advantages for surgeon and patient. Compared to moderate NMB, deep NMB produced stable and improved surgical conditions with less postoperative pain.

## Introduction

In a previous study we showed that administration of muscle relaxants to the level of a deep neuromuscular blockade (NMB) significantly improves the quality of the surgical field during retroperitoneal laparoscopic surgery [1]. Using the 5-point Leiden-Surgical Rating Scale (L-SRS; Table 1) ranging from 1 (extremely poor conditions) to 5 (optimal conditions), we observed that during moderate NMB (defined by a 1–2 thumb twitch response to the train-of-four stimulation of the ulnar nerve) 20% of scorings were in the low range (L-SRS of 1 to 3) while during deep NMB (0 twitch count in the train-of-four, 1–2 twitch responses in the post-tetanic count) 99% of scorings were in the range 4 to 5 [1]. Importantly, due to the availability of an effective and rapid-acting reversal agent, the deep NMB could be maintained until the end of surgery without negatively affecting postoperative conditions. Although the surgical team felt that the differences in L-SRS (a mean difference of 18%) were highly clinically relevant, the study was criticized because of its limited size and the lack of important differences in patient outcome or incidence of adverse events [2]. Studies on the effect of a deep NMB on patient outcome are highly relevant and a positive difference in outcome (such as a difference in postoperative pain) will further justify the use of deep NMB in laparoscopic surgeries. So far, none of the studies comparing deep and moderate NMB observed a difference in postoperative outcome (see Ref. [3] and references cited therein).

**Table 1:** The Leiden Surgical Rating Scale (L-SRS).

**1.**	**Extremely poor conditions:** The surgeon is unable to work due to coughing or due to the inability to obtain a visible laparoscopic field because of inadequate muscle relaxation. Additional muscle relaxants must be given.
**2.**	**Poor conditions:** There is a visible laparoscopic field but the surgeon is severely hampered by inadequate muscle relaxation with continuous muscle contractions and/or movements with the hazard of tissue damage. Additional muscle relaxants must be given.
**3.**	**Acceptable conditions:** There is a wide visible laparoscopic field but muscle contractions and/or movements occur regularly causing some interference with the surgeon’s work. There is the need for additional muscle relaxants to prevent deterioration.
**4.**	**Good conditions:** There is a wide laparoscopic working field with sporadic muscle contractions and/or movements. There is no immediate need for additional muscle relaxants unless there is the fear for deterioration.
**5.**	**Optimal conditions:** There is a wide visible laparoscopic working field without any movement or contractions. There is no need for additional muscle relaxants.

To assess whether our previous findings can be extrapolated to other types of laparoscopic surgery, we performed a double blind controlled randomized trial comparing deep *versus* moderate NMB on differences in surgical working conditions during bariatric laparoscopic surgery. Additionally we addressed the issue of patient outcome and powered the study to detect a difference of deep *versus* moderate NMB on pain scores in the post-anesthesia care unit (PACU) and on the ward.

We hypothesize that, relative to a moderate NMB, a deep NMB improves the quality of the surgical field and results in lower postoperative pain scores.

## Methods

### Ethics Statement and Trial Registration

The study including follow-up was carried out between 1 September 2015 and 30 April 2016, after approval was obtained from the ethics committee of the Leiden University Medical Center and Haaglanden Medical Center. The protocol was registered at trialregister.nl under identifier NTR5380. Patients scheduled to undergo elective Roux-Y-Gastric Bypass (RYGB) surgery were approached at least one week before surgery and received oral and written information about the study. All patients that indicated willingness to participate gave written informed consent prior to enrolment. All data capturing was performed according to Good Clinical Practice guidelines. The protocol is available for review (see S1 Protocol).

### Patients

ASA 1–3 patients with age 18 to 65 years of either sex, scheduled to undergo RYGB surgery, were recruited to participate in the study. Exclusion criteria were inability to give informed consent, known or suspected allergies to medication used during anesthesia, a (family) history of neuromuscular disease, a (family) history of malignant hyperthermia and renal insufficiency (defined by a glomerular filtration rate < 30 mL/min).

### Monitoring and Study Design

#### Anesthesia

All patients received total intravenous anesthesia with propofol, remifentanil and rocuronium. Propofol and remifentanil were delivered by continuous infusion, rocuronium by bolus administrations. Monitoring was according to local practice and consisted of electrocardiography, blood pressure, heart rate and bispectral index monitoring using the Philips BIS module system (Philips NV, Amsterdam, The Netherlands). BIS values were kept between 40 and 60 throughout anesthesia.

#### Neuromuscular monitoring

Neuromuscular function was monitored using acceleromyography with the TOF-watch-SX monitor (MSD BV, Haarlem, The Netherlands). To that end two electrodes were placed over the ulnar nerve of the left or right wrist, the ipsilateral thumb was placed in a flexible adaptor to generate preload and a sensor was placed on the tip of the thumb. Adduction of the thumb through contractions of the adductor pollicis brevis muscle were detected by the sensor on the thumb. After induction of anesthesia but prior to any administration of rocuronium, the device was calibrated according to the specification of the manufacturer (see also Ref. 1 for an explanation). In the study we measured the thumb twitch response to four subsequent electrical stimuli (*i*.*e*. the train-of-four or TOF) at 10 min intervals. In case zero thumb twitches were detected the post-tetanic count (PTC) was measured.

#### Treatment levels

Patients were randomized to a moderate neuromuscular block (NMB) or a deep NMB. Patients received intravenous rocuronium 30 mg prior to intubation followed by repeated 10 mg intravenous doses to reach a TOF count of 1–2 twitches (moderate NMB) or 0 twitches and 2–3 twitches in the PTC (deep NMB). The initial rocuronium dose is the standard first dose used in obesity surgery in our clinics.

#### The Leiden-Surgical Rating Scale (L-SRS)

During the procedure, the L-SRS was scored at 10-min intervals by the surgeon performing the RYGB surgery (DS, MK, RL). The scoring system was first practiced during several procedures not included in the study. The L-SRS is a Likert scale ranging from 1 to 5 where 1 indicates *extremely poor conditions*, 2 *poor conditions*, 3 *acceptable conditions*, 4, *good conditions* and 5 *optimal* conditions [1]. See Table 1 for a further explanation of the scoring system. The L-SRS aims to quantify the quality of the surgical field based on visibility, surgical space, muscle contractions, handling tactics and patient movement. In two previous studies we used L-SRS in retroperitoneal surgery and observed consistent L-SRS scorings depending on the depth of NMB without any effect of other factors such as duration of surgery, ventilator settings and level of arterial PCO_2_ [1,4].

#### Reversal

At the end of surgery, a moderate NMB was reversed with sugammadex (MSD BV, Haarlem, The Netherlands) 2 mg/kg ideal body weight, while a deep NMB was reversed with sugammadex 4 mg/kg ideal body weight [5]. The endotracheal tube was removed when the TOF-ratio > 0.9 and the patient breathed spontaneously and was fully awake.

#### Postoperative pain measurement and treatment

Pain was measured four times in the PACU (at 10 min intervals starting upon arrival) and twice on the ward (8PM the day of surgery and 2 PM the next day). On the ward three distinct types of pain were queried: superficial and deep wound pain and referred shoulder pain. Pain was measured using an 11-point numerical rating scale (NRS), ranging from 0 (no pain) to 10 (most pain imaginable).

The handling of postoperative pain was initially based on preemptive treatment with paracetamol (1000 mg) and Celebrex 200 mg, both given preoperatively, and intravenous piritramide 10 mg given after induction of anesthesia. In the post-anesthesia-care unit (PACU) all patients received intravenous piritramide when the pain score was greater than 4, followed by patient controlled analgesia with morphine (IV MPCA). The IV MPCA pump was set to deliver 1 mg morphine with a lockout of 7 min; no background infusion was allowed. Additionally, the patients received oral paracetamol 1 gram every eight hours and oral Celebrex 200 mg upon arrival on the ward. All patients were discharged 24 h after arrival on the ward.

To assess the treatment effect on the combination of pain data and analgesic medication, we used a composite score that was calculated by averaging the 4 NRS scores in the PACU and adding the average score to each mg of morphine or piritramide used in the PACU. Composite scores similar to ours have been shown previously to enable identification of analgesic treatment benefit and enhance the sensitivity of assessment of an intervention [6,7].

### Data Management

#### Measurements

The following variables were collected: patient variables (age, weight, height, sex, previous pregnancies, ASA classification), anesthesia-related variables (drug dosages, BIS, time from reversal until extubation, ventilator settings, TOF, PTC, TOF-ratio), hemodynamic variables (blood pressure, heart rate), surgery-related variables (L-SRS, duration of the procedure), and variables obtained in the PACU and on the ward (pain in the PACU, occurrence of nausea/vomiting, breathing rate, oxygen saturation, sedation level, opioid consumption, deep and superficial wound pain on the ward, referred shoulder pain on the ward). The averages of BIS, hemodynamic and respiratory variables obtained during surgery are reported.

#### Blinding and randomization

The study had a double blind design with the surgeons, the research team, and PACU and ward nurses fully blinded to treatment. Only the attending anesthesiologist and anesthesia nurses were unblinded and were responsible for the administration of the correct NMB. Randomization was performed just prior to induction of anesthesia; the attending anesthesiologist received the randomization code at induction from an independent individual that was not part of the research team.

#### Sample size calculation

We initially powered the study to detect a difference in L-SRS between treatments of 0.5 points with SD 0.4. A sample size of 20 per groups was calculated which would provide at least 90% power to detect the expected difference in L-SRS at α = 0.05 [4]. However, we additionally designed the study to detect a difference in pain score in the PACU. The *a priori* estimated mean difference between the treatment groups was 1 point on the 10-point NRS scale. Assuming a standard deviation of 1.5, a sample size of 47 subjects per group would provide at least 90% power to observe the expected difference at alpha = 0.05. A sample size of 50 per group was chosen to take into account any margin of uncertainty around the effect size and SD. The sample size calculation was performed in SigmaPlot v12 (Systat Software Inc., San Jose, CA).

#### Statistical analysis

There were two main end-points: L-SRS during surgery and postoperative pain scores (NRS). The L-SRS and the pain scores in the PACU were compared using a linear mixed effects model with ARMA1 (autoregressive moving average covariance structure) correction. To estimate the variability (95% confidence interval) bootstrap analyses were performed. Comparisons of scores at fixed times points were done with Mann-Whitney-U tests. The two pain scores obtained on the ward were averaged per patient. Treatment effects on the mean ward NRS scores and on the composite score were tested using Mann-Whitney-U tests. Statistical analysis was performed using the SPSS statistical software package (version 23.0; SPSS Inc., Chicago, IL). P-values < 0.05 were considered significant. Values are mean (95% confidence interval) unless otherwise stated).

### Comparison of the L-SRS among Surgeons

To get a first indication of the utility of our scoring system we compared the L-SRS scores between surgeons. Since there is no gold standard or other validated rating scale to score surgical conditions against which we can compare our scoring system, we reasoned that our L-SRS is useful if at increasing levels of relaxation the L-SRS increases. We expanded the current data set with 50 additional RYGB patients that received a single administration of rocuronium 30 mg at induction on top of propofol/remifentanil anesthesia (further referred to as single NMB). To assess whether the scoring of the surgeons was similar amongst levels of NMB we performed a two-way analysis of variance (factors: surgeon, treatment (NMB) and surgeon**×**treatment) and determined the within-observer variability.

## Results

**Fig 1:**
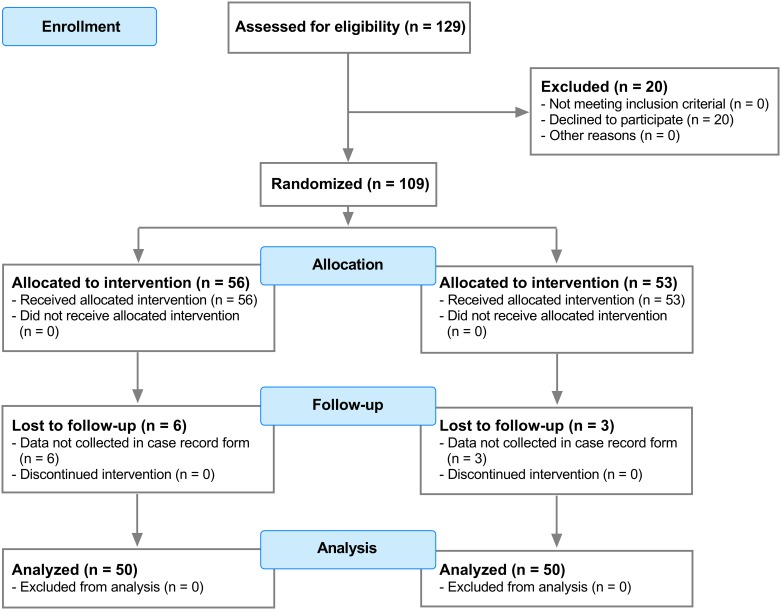
Consort flow diagram.

A total of 109 patients were enrolled in the study, randomized and received treatment according to protocol (Fig 1). Nine patients (3 in the moderate NMB and 6 in the deep NMB group) dropped out of the study because of logistic reasons, most importantly because the study variables were not inputted into the electronic case record form. All nine were replaced in a fully blinded fashion. Patient characteristics are given in Table 2, showing no differences between study groups. All patients were morbidly obese with BMI values greater than 35 kg/m^2^.

**Table 2:** Patient characteristics.

	Moderate NMB block	Deep NMB block
Number of patients	50	50
Females (*n*)	41 (82%)	39 (78%)
Previous pregnancy in females (*n*)	34 (83%)	25 (64%)
Age (years)	46.9 ± 10.6	47.2 ± 11.1
Weight (kg)	124 ± 21	127 ± 20
Height (cm)	169 ± 10	171 ± 9
Body mass index (kg/m^2^)	43.0 ± 4.5	43.3 ± 5.1
ASA classification (*n*)		
1	-	-
2	46 (92%)	42 (84%)
3	4 (8%)	8 (16%)

Values are mean ± SD or numbers (*n* (%)).

The two study groups were comparable with respect to duration of surgery and anesthetic and opioid administration (Table 3). A total median (range) rocuronium dose of 40 mg (30–130) resulted in a moderate NMB with an average number of TOF-twitches (95% confidence interval) of 1.9 (1.7–2.1), which is within the target range of 1–2. A median (range) dose rocuronium of 70 mg (45–145) was needed to induce a deep NMB with zero twitches. The average number of twitches in the PTC was 3.6 (3.2–4.0), which is somewhat higher than the target range of 2–3 (Table 3). Ventilator settings, peak inspiratory flow, intra-abdominal pressure and hemodynamics did not differ between moderate and deep NMB during anesthesia.

**Table 3:** Measurements obtained during and following surgery.

	Moderate NMB block	Deep NMB block	p-value
**Anesthesia**			
Duration of surgery (min)	41 (24–115)	43 (24–78)	
Propofol dose (mg)	438 ± 120	460 ± 100	
Remifentanil dose (mg)	1.0 ± 0.4	1.1 ± 0.4	
Rocuronium induction dose (mg)	30 (30–30)	30 (30–30)	
Rocuronium total dose (mg)	40 (30–130)	70 (45–145)	
Piritramide dose (mg)	10 (10–10)	10 (10–10)	
Bispectral index of the EEG	42 ± 7	43 ± 5	
Number of twitches TOF	1.9 ± 0.6	0 (0–0)	
Number of twitches PTC	n.a.	3.6 ± 1.4	
L-SRS^#^	4.2 [4.0–4.4]	4.8 [4.7–4.9]	< 0.001
Intra-abdominal pressure (cm H_2_O)	18 (0)	18 (0)	
Mean blood pressure (mm Hg)	86 ± 13	85 ± 14	
Heart rate (min^-1^)	83 ± 12	81 ± 13	
Inspiratory volume (mL)	573 ± 59	579 ± 65	
Ventilator rate (min^-1^)	14 (10–15)	14 (12–16)	
Peak inspiratory pressure (mm Hg)	24 ± 2	25 ± 2	
**Reversal**			
Sugammadex dose (mg)	132 (100–200)	266 (180–370)	
Time to extubation (min)^$^	3 (1–6)	3 (1–8)	
**Post-Anesthesia Care Unit**			
Pain score (NRS)^#^	4.4 [4.2–4.9]	3.9 [3.6–4.4]	0.03
IV M PCA mg	4 (0–8)	3 (0–10)	
Piritramide (mg)	24 (10–25)	20 (10–25)	
Respiratory rate (min^-1^)	12.7 ± 0.7	12.6 ± 1.1	
Sedation score*	2.3 (1.8–3.3)	2.3 (1.8–4.0)	
Oxygen saturation (%)	98.4 ± 1.6	98.5 ± 1.6	
Mean blood pressure (mm Hg)	98 ± 13	101 ± 15	
Heart rate (min^-1^)	73 ± 13	70 ± 11	
Nausea (*n*)	25 (50%)	27 (54%)	
**On the ward**			
Superficial wound pain score (NRS)^#^	1.9 [1.7–2.1]	1.6 [1.4–1.8]	NS
Deep wound pain (NRS) ^#^	2.3 [2.0–2.6]	1.8 [1.5–2.1]	NS
Referred shoulder pain (NRS) ^#^	1.8 [1.5–21]	1.3 [1.1–1.5]	0.03

^$^ from the time of reversal;

* 5-point scale from 0 (no sedation) to 5 (not arousable); NRS numerical rating score; n.a. not available; Hemodynamic, BIS and respiratory data are averages of the 1-min data collected in the electronic patient data system during surgery.

Values are mean of the patients’ mean ± SD, median (range), numbers (*n* (%)) or

^#^ mean [95% confidence interval]; NS not significantly significant

### The Leiden-Surgical Rating Scale during Bariatric Surgery

**Fig 2:**
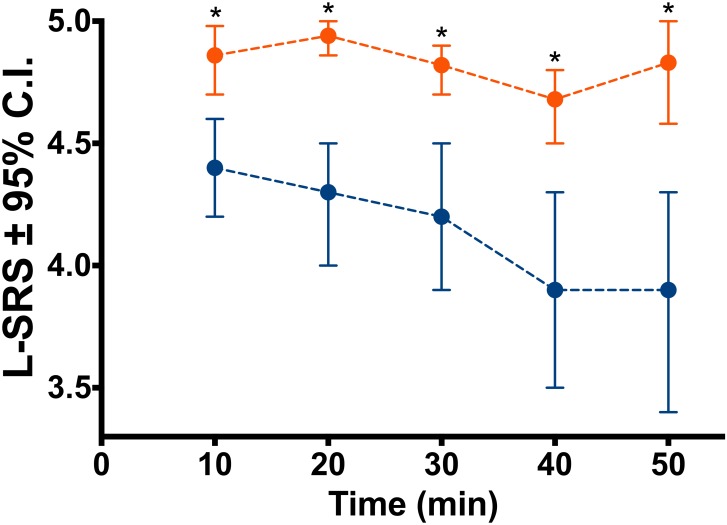
Influence of deep relaxation on surgical conditions. Leiden-surgical rating scale (L-SRS) values of patients during a deep neuromuscular block (target PTC 2–3; orange, *n* = 50) and during a moderate neuromuscular block (target TOF 1–2; blue, *n* = 50). Values are mean (95% confidence interval) with confidence intervals derived from bootstrap analyses. * Mann-Whitney-U test p < 0.01 *versus* moderate block.

**Fig 3:**
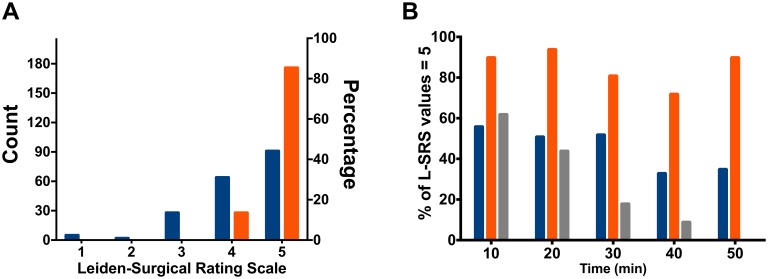
Distribution of L-SRS scores. **A**. Distribution of the individual Leiden-surgical rating scale (L-SRS) scores across the 5 possible categories (see Table 1). **B**. Percentage of L-SRS values of 5 over time in single, moderate and deep blocks. Orange depicts deep NMB, blue moderate NMB and grey single NMB.

We obtained 208 ratings in the 50 patients that received a moderate block and 204 ratings in the 50 that received a deep block. The L-SRS ratings differed significantly between moderate and deep NMB. Mean L-SRS scores in moderate NMB were 4.2 (95% confidence interval 4.0–4.4) *versus* 4.8 (4.7–4.9) (p < 0.001). In Figs 2 and 3 we give the mean scores (95% confidence interval) at each time point, the distribution of individual scores across the 5-points of the L-SRS (Fig 3A) and the percentage of scores at an L-SRS of 5 (Fig 3B). The three panels show the lack of poor conditions in the deep NMB in contrast to the moderate block. In the moderate block, at all times points a score of 3 or less was observed; in the deep block 100% of scorings during deep NMB were in the range 4–5 (good and optimal conditions). Consequently the variability in scores was higher for the moderate NMB (17%) than deep NMB (4%).

A majority of women studied had previous pregnancies (moderate NMB 83%, deep NMB 64%). Since these may affect the muscular strength of the abdominal wall we performed a separate analysis on this population. The mean L-SRS (95% confidence interval) in moderate NMB (n = 34) was 4.1 (3.8–4.5) against deep NMB (n = 25) 4.8 (4.7–4.9) (p = 0.02).

### Measurements in the PACU and on the Ward

The pain scores in the PACU differed significantly between NMB levels with a 0.7 higher NRS following moderate compared to deep NMB (moderate NMB 4.4 (95% confidence interval: 4.2–4.9) *versus* deep NMB 3.9 (3.6–4.4), p = 0.03; Table 3). Analgesic medication did not differ between treatment groups although there was a tendency towards less medication use in patients after deep NMB. Combining NRS scores and medication into the composite score resulted in large difference between treatments (moderate NMB 33 (31–35) *versus* deep NMB 27 (26–28), p = 0.001).

On the ward no difference in superficial and deep wound pain was observed but referred shoulder pain was 0.5 point less in patients that had received a deep NMB (moderate NMB 1.8 (1.5–2.1) *versus* 1.3 (1.1–1.5), p = 0.03; Table 3), although all scores were on the lower end of the NRS (< 3), which suggest a clinically less relevant difference. No difference in opioid consumption on the ward was present between moderate and deep NMBs (data not shown).

None of the other variables obtained in the PACU (hemodynamics, respiration, sedation, nausea) differed between moderate and deep NMBs (Table 3).

### Comparison of the Leiden-Surgical Rating Scale among Surgeons

The added population of 50 patients (37 women/13 men) were similar in characteristics compared to the 100 patients in the randomized study: 46 patients had an ASA II classification, the four others an ASA III classification; age 46 ± 9 years; weight 122 ± 14 kg; height 160 ± 10 cm; BMI 43 ± 4.6 kg/m^2^ (values are mean ± SD). The number of twitches in the TOF ranged from 2 to 4 over time with an average TOF ratio ranging from 0.22 to 0.60 when the TOF count was 4; the mean L-SRS was 4.1 (4.0–4.3). The percentage of scores at an L-SRS of 5 at different time points is given in Fig 3B.

The intra-observer variability was the lowest at the deep NMB with similar intra-observer variabilities across surgeons independent of the level of relaxation: single NMB 11, 12 and 19% for surgeon A, B and C, respectively; moderate NMB 12, 16 and 19%; and deep NMB 3, 4 and 6% (values are % coefficient of variation). The analysis of variance revealed significant effects for surgeon (p = 0.03), treatment (p < 0.001) but not for surgeon**×**treatment (p = 0.76), indicative that the three surgeons had similar increasing scores at increasing levels of relaxation.

## Discussion

We here show for the first time that deep relaxation, compared to moderate relaxation, improves surgical conditions by 0.7 points during laparoscopic bariatric surgery coupled to lower pain scores in the PACU and less referred shoulder pain in the ward.

Until recently the use of a deep NMB was hindered by long recovery times and postoperative complications, such as incomplete reversal of NMB with compromised respiratory and upper airway functions. The recent introduction of sugammadex makes rapid reversal of a deep NMB possible without concerns for postoperative complications due to persistent relaxation [8]. Sugammadex is a modified γ-cyclodextrin that selectively binds rocuronium and vecuronium in plasma and consequently causes the rapid diffusion of these relaxants away from the neuromuscular junction and thus induces rapid and intense reversal of the NMB [9]. Indeed, the introduction of sugammadex made the current study practically feasible without any concession to postoperative patient conditions and with acceptable turnover times (Table 3).

### Deep Neuromuscular Block and Outcome

The value of a deep NMB on benefits for surgeon and patient has recently been debated [10,11]. It was argued that objective data are missing to support the idea that a deep NMB contributes to improved surgical operating conditions and a better patient outcome [10]. In one of the largest studies so far, we here show that both surgical conditions and patient outcome are improved following deep *versus* moderate NMB conditions. Our data are in agreement with a previous systematic review on this topic [12]. The question remains whether the observed changes in mean L-SRS of 17% and reduction of NRS from 4.6 to 3.9 are clinically relevant. Only the surgical team can address the first item. All three surgeons involved in the study considered the change in L-SRS important and clinically relevant. With respect to the second item, we believe that the reduced pain scores in the PACU and on the ward, albeit modest in magnitude, is a highly important and clinically relevant observation. It may well be that reducing the intra-abdominal pressure will further reduce postoperative pain scores and possibly also postoperative opioid consumption (see below).

The cause of pain following laparoscopic surgery and the pneumoperitoneum in the morbidly obese is complex and various factors may be involved including tissue damage, high insufflation pressures, activation of peritoneal nociceptors by carbon dioxide (particularly in case of residual CO_2_), reduced abdominal muscle compliance, duration of surgery and production of pro-inflammatory cytokines in white adipose tissue. Although our study was not designed to study the mechanism through which a deep NMB reduces the pain scores postoperatively, it is of importance to discuss possible mechanisms. We do not believe that the use of sugammadex *per* se had an effect on pain scores in our study as both treatment arms received sugammadex. Castro and coworkers compared sugammadex with neostigmine reversal following bariatric surgery (we remain uninformed on the depth of NMB during surgery) and observed a significant difference in pain scores in the PACU in favour of sugammadex (23% of patients had a pain score ≥ 4 after sugammadex *versus* 59% after neostigmine, p < 0.05) [13]. Possibly in our study the higher sugammadex dose given to patients with a deep NMB contributed to the lower pain scores.

It has been hypothesized that low insufflation pressures during laparoscopic surgery may contribute to less postoperative pain, especially less shoulder pain.^3^ There is some proof for this in the literature [14,15] (but see Refs. [3,16]). In our current study the intra-abdominal pressure was kept constant at 18 cm H_2_O in both treatment arms, as is commonly used in bariatric pressure [17]. Although this implies that the intra-abdominal pressure could not contribute to our observations of less postoperative pain, it may well be that at deep relaxation the tension on the abdominal wall exerted by the intra-abdominal pressure was less harmful in comparison to the tension created during moderate relaxation.

### Critique

We used propofol to induce general anesthesia in this study, as well as in our previous studies [1,4]. Possibly the use of inhalational anesthetics might have resulted in a different outcome as additional relaxation is expected from volatile anesthetics. Further studies are needed to address this important issue.

In this study, the deep NMB was obtained at PTC values of 3.6, somewhat higher than the target range of 2–3. We remain unaware whether this influenced the outcome of our study but assume that a stricter regimen towards lower PTC values would have further improved L-SRS scores in the deep NMB.

As may be observed in Fig 2, at t = 40 min, L-SRS values decreased during surgeries under moderate NMB. This was not related to any change in level of relaxation (mean TOF remained constant at 2 twitches). We relate this to the more complex part of surgery (jejuno-jejunostomy) with potentially some interference of the omentum or other fatty structures. A small decrease in L-SRS was also present at t = 40 min in the deep NMB but the effect was less prominent.

It is important to realize that surgical conditions may be improved by other measures than just deep NMB [1]. For example, regional anesthesia, high concentrations of intravenous or inhalational anesthetics, high dose opioids and/or hypocapnia may possibly reduce muscle activity and consequently abdominal wall tension, especially when these methods are combined. So far, we were unable to observe an effect from hypocapnia on the L-SRS [4]. Further studies are needed to assess the effect of these methods and their combination on patient outcome and recovery times.

### Validation of the Leiden-Surgical Rating Scale (L-SRS)

Although others have used surgical rating scales [18–20], there is no gold standard on how to rate surgical conditions during laraproscopic surgery. We developed a 5-point rating scale that has already been used in several studies [1,4,21,22]. Additionally, screening the US National Institutes of Health clinical registry (clinicaltrial.gov) showed that various ongoing trials also use the L-SRS (see for example, NCT02320734, NCT02601508 and NCT02888067). We argue that the L-SRS is a useful tool, but one that awaits formal validation. Some initial proof of its validity is given here.

1. In the current study, good and optimal conditions were observed in 83% of scorings during moderate NMB and 100% during deep NMB. These finding are in close agreement with previous observations in a very different type of surgery (retroperitoneal prostate and renal surgery) with good and optimal conditions in 82% and 99% of scorings in moderate and deep blocks, respectively [1]. Additionally, both studies show a similar degree of variability in ratings with a high variability for the moderate NMB (17–26%) and low variability for the deep NMB (4%). These data indicate that the use of the L-SRS yields highly reproducible results between different surgical laparoscopic interventions.
2. We further compared the results of the three independent surgeons involved in the current study. We added a cohort of 50 RYGB patients that received a single dose of rocuronium upon induction of anesthesia, which produced a moderate to shallow NMB. This approach allowed us to assess the scoring of three levels of NMB with TOF values of 3.2, 1.9 and 0. For all three surgeons, an increase in L-SRS at an increasing depth of NMB was observed with similar absolute scorings and intra-observer variabilities.
3. We previously collected video snippets of the surgical field obtained concurrently with L-SRS scorings in retroperitoneal urological surgery [1]. The video images were later scored by 12 senior anesthesiologist and 8 surgeons specialized in laparoscopic surgery for gastrointestinal procedures. As we expected, there was little agreement between L-SRS scorings of the anesthesiologist and the urologist (kappa-statistic 0.05). In contrast, the agreement was much greater between the specialized surgeons and the urologist (kappa-statistic 0.5).
4. In another cohort of patients that underwent retroperitoneal laparoscopic surgery we studied whether the L-SRS is sensitive to other factors such as arterial PCO_2_ and ventilator settings [4]. Hypocapnic conditions (arterial PCO_2_ 25.5 mm Hg) with ventilator settings of 8 mL/kg and rate 20/min were compared to hypercapnic conditions (arterial PCO_2_ 42.8 mm Hg) with ventilator settings of 8 mL/kg and rate 12/min. We observed similar and stable ratings over time during deep relaxation (average L-SRS scores 4.8 in both groups).
5. Other independent investigators have used the L-SRS successfully in robotic laparoscopic prostate surgery and microlarynx surgery comparing deep *versus* moderate NMB [21,22]. In both of these studies the scorings during deep and moderate NMB were similar to those observed in our studies.
6. Finally, comparing L-SRS scores at similar levels of NMB across studies shows that the L-SRS is independent of body mass index (this study *versus* Refs. [1,4]). Additionally in this study we show that the L-SRS is independent of previous pregnancies.

Given the above, the data presented here give ample proof of the ability of the L-SRS to satisfactorily quantify the surgical work field. The L-SRS seems sensitive to the level of NMB without interference of outside factors such as variations in arterial PCO_2_, ventilator settings, surgeon, type of surgery and patient characteristics. Further independent studies are required to assess the formal validity of the L-SRS.

### Conclusions

We show that in bariatric laparoscopic surgery deep *versu*s moderate neuromuscular blockade produced stable and improved surgical conditions with less pain in the PACU and less shoulder pain on the ward. Further studies are underway studying the relationship between depth of the NMB, intra-abdominal pressure and patient outcome, including pain, hemodynamics and pulmonary function. The improved working conditions during deep NMB is for our surgeons of high clinical importance and consequently a significant factor for the application of this technique in certain but definitely not all laparoscopic surgeries in our clinic.

## Supporting Information

S1 CONSORT Checklist (DOC)

S1 File This file contains the raw data set. (XLSX)

S1 Protocol This is the study protocol. (PDF)
