# Supplementary material for: Deep Neuromuscular Block Improves Surgical Conditions during Bariatric Surgery and Reduces Postoperative Pain: A Randomized Double Blind Controlled Trial
Source: PLoS One. 2016 Dec 9;11(12):e0167907. doi: 10.1371/journal.pone.0167907 (PMC5148011; doi:10.1371/journal.pone.0167907)
Supplement: S1 Protocol — (PDF) [file pone.0167907.s003.pdf]

Protocol associated with
"A novel anesthesia technique that improves surgical conditions during bariatric surgery and reduces postoperative pain: a randomized double blind controlled trial"

Protocol date: March 12 – 2015   Approval from Ethics: June 16 - 2015

Address for correspondence
Albert Dahan MD PhD, Professor of Anesthesiology
Department of Anesthesiology, Leiden University Medical Center
Albinusdreef 2, 2333 ZA Leiden, The Netherlands
TEL +31 71 526 2301 | FAX +31 71 526 6230 | E-mail a.dahan@lumc.nl

1. Background

In laparoscopic surgery, especially when surgery is performed in morbidly obese patients, surgical conditions are determined in a major if not exclusive part by the depth of the neuromuscular block. A deep block (PTC 1-2) is often associated with improved surgical conditions and is therefore requested by the surgeons. However, a deep block comes at the expense of a variety of items that may conflict with its use including long recovery times, postoperative ventilation and impaired postoperative breathing conditions with atelectasis and hypoxia. With the introduction of Sugammadex there is now the possibility to reverse an even deep surgical block. This may overcome most of the issues mentioned.

Previously we showed that a deep NMB coincided with favorable surgical conditions in lean patients undergoing elective laparoscopic retroperitoneal surgery for prostatectomy or nephrectomy (See Figure 1). During a moderate NMB 20% of surgical scorings were less than good, during deep NMB 99% of scorings were excellent or good, 67% were excellent.

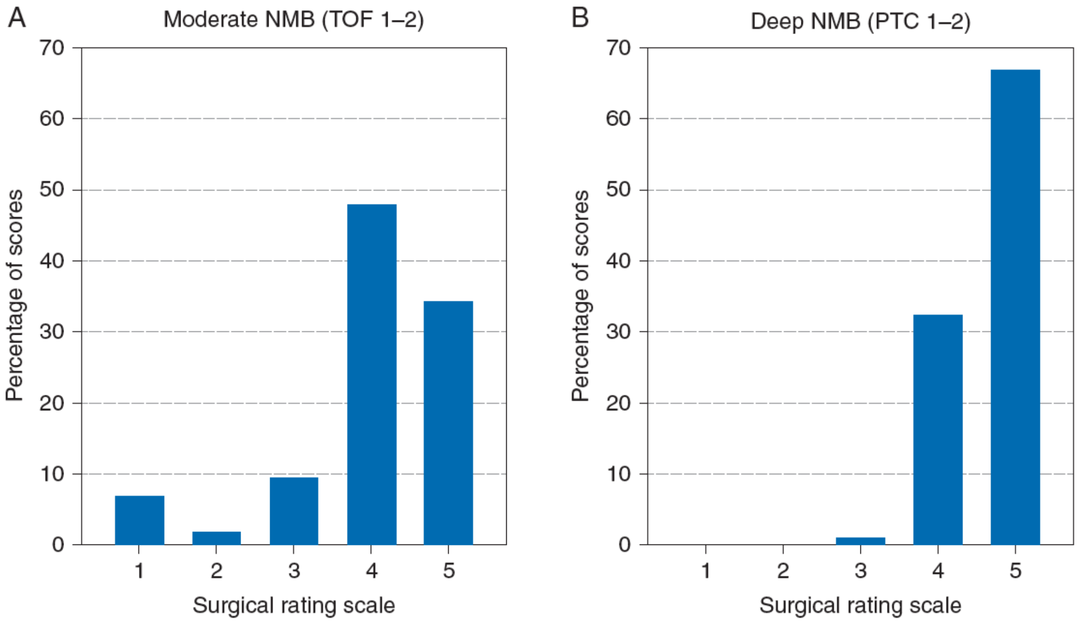

Figure 1: Effect of depth of NMB of scoring of the surgical field using the Leiden Surgical Rating Scale in lean patients undergoing retroperitoneal urologic surgery. The scores range from 1 (extreme poor conditions) to 5 (excellent conditions). Data are from Martini et al. 2014.

In the current study we will examine the effect of the depth of the neuromuscular block on two major end-points:

(1) Surgical conditions, with as main research question "*Does a deep surgical block indeed improve surgical conditions in case of laparoscopic surgery in morbidly obese patients for bariatric surgery?*" The surgical condition will be determined from a surgical rating condition scale, the Leiden Surgical rating Scale. This is a 5-point ordinal scale ranging from 1 = poor condition to 5 = optimal surgical conditions. The surgeon will score the condition at 5 min intervals. At the time of scoring also the intraabdominal pressure will be monitored.
(2) Postoperative pain, with as main question "*"Does a deep block coincide with less pain compared to a moderate block in the recovery period?"*

Secondary end-points include:
(1) The hemodynamics during surgery with the main research question "*Does a deep neuromuscular block coincide with improved hemodynamics during surgery and less postoperative pain?"*
(2) Cardiorespiratory conditions in the post-anesthesia care unit, with the main research question "*What is the effect of reversal with Sugammadex of a deep surgical muscle block on postoperative breathing activity as measured by respiratory rate and saturation*"
(3) Sedation (Using the Ramsey Sedation Scale) in the post-anesthesia care unit, with the main research question *"Does a deep block coincide with less sedation compared to a moderate block in the recovery period?"* and
(4) The Postoperative Quality of Recovery Scale (PQRS), with the main question *"Irrespective of depth of the neuromuscular block during surgery, is the quality of recovery similar in patients that were reversed with sugammadex?".*

To address these research questions, we will relax morbidly obese patients undergoing bariatric surgery with rocuronium. Patients will be randomly assigned to receive a moderate neuromuscular block with a TOF of 1-2 or a deep NMB (deep block) with a TOF of zero and PTC of 1-2. Rocuronium will be administered as bolus infusion ranging from 0.6 – 1 mg/kg. Titration to effect will be performed immediately after intubation.

After surgery has ended patients that received a moderate NMB will be reversed with 2 mg/kg sugammadex, while patient that received the deep block will receive 4 mg/kg sugammadex. Extubation will be performed when the TOF ratio > 0.9 and the patients breathes adequately.

In the post-anesthesia care unit the cardiorespiratory parameters (blood pressure, heart rate oxygen saturation), the level of pain (on an 11-point numerical rating scale), sedation and the PRQS will be measured at 15 min intervals until discharge to the ward.

2. Objectives and Hypotheses

Main Objectives

- To assess whether the implementation of a deep neuromuscular block (NMB) (PTC 1-2) combined creates optimal surgical conditions as measured by the surgeon (using the 5-point Leiden surgical rating scale) *versus* a moderate neuromuscular block (TOF 1-2) in morbidly obese patients undergoing bariatric surgery.
-To assess whether reversal of the deep NMB with sugammadex 4 mg/kg results in optimal analgesic conditions in the post-anesthesia care unit.

Hypothesis

-Deep NMB combined will result in a significant improvement of surgical conditions compared to moderate NMB, combined with less pain postoperatively;

3. Study Design

This is a randomized controlled trial (RCT) performed in 100 morbidly obese patients that will undergo elective laparoscopic surgery for bariatric surgery under a moderate neuromuscular block (GROUP 1) or a deep neuromuscular block (GROUP 2). All procedures will be executed in compliance with the current revision of the Declaration of Helsinki and Good Clinical Practice guidelines. The trial starts after the medical ethical committee has approved the study protocol and will have a maximum duration of one year.

Inclusion criteria: ASA 1-3, 18 years or older and younger than 66 years; BMI > 34 kg/m^2; ability to give informed consent; elective bariatric surgery.

Exclusion criteria: known or suspected neuromuscular disorders impairing neuromuscular function; allergies to muscle relaxants, anesthetics or narcotics; a (family) history of malignant hyperthermia; women who are or may be pregnant or are currently breast feeding; renal insufficiency, as defined by serum creatinine x 2 of normal, or urine output < 0.5 ml/kg/h for at least 6 h. When available, other indices will be taken into account as well such as glomerular filtration rate < 30 ml/h and proteinuria (a ratio of 30 mg albumin to 1 g of creatinine).

Patients in GROUP 1 will receive intravenous rocuronium bolus infusions until the TOF = 1 or 2; patients in GROUP 2 will receive intravenous rocuronium bolus infusions until the TOF = 0 and PTC = 1 or 2. The TOF will be measured by TOF watch at 5 min intervals allowing titration of the neuromuscular block to effect. This procedure is similar to that of Dubois et al. (Eur J Anaesthesiol, 2014). **The surgeon will be blinded to the level of muscle relaxation.** The attending anesthesiologist will be made responsible for both the administration of the muscle relaxant and degree of NMB. The anesthesia consists of propofol plus remifentanil aimed at keeping the BIS at a value of 50 ± 10.

During surgery the surgeon will score the surgical conditions according to the Leiden Surgical Rating Scale (Martini et al., 2014): The score are defined as follows:

**1**	**Extremely poor conditions:** The surgeon is unable to work due to coughing or due to the inability to obtain a visible laparoscopic field because of inadequate muscle relaxation. Additional muscle relaxants must be given.
**2**	**Poor conditions:** There is a visible laparoscopic field but the surgeon is severely hampered by inadequate muscle relaxation with continuous muscle contractions and/or movements with the hazard of tissue damage. Additional muscle relaxants must be given.
**3**	**Acceptable conditions:** There is a wide visible laparoscopic field but muscle contractions and/or movements occur regularly causing some interference with the surgeon’s work. There is the need for additional

	muscle relaxants to prevent deterioration.
4	**Good conditions:** There is a wide laparoscopic working field with sporadic muscle contractions and/or movements. There is no immediate need for additional muscle relaxants unless there is the fear for deterioration.
5	**Optimal conditions:** There is a wide visible laparoscopic working field without any movement or contractions. There is no need for additional muscle relaxants.

After the surgery has finished patients will be reversed with sugammadex: GROUP 1 will receive 2 mg/kg, GROUP 2 4 mg/kg. This will be known by the attending anesthesiologist only and will be blinded to surgeon and the PACU team.

In the recovery room (post-anesthesia care unit) the following measurements are made at 10 min interval: blood pressure, heart rate, respiratory rate, SpO_2, pain, PQRS and sedation levels by the recovery room personnel, who all will be blinded to treatment.

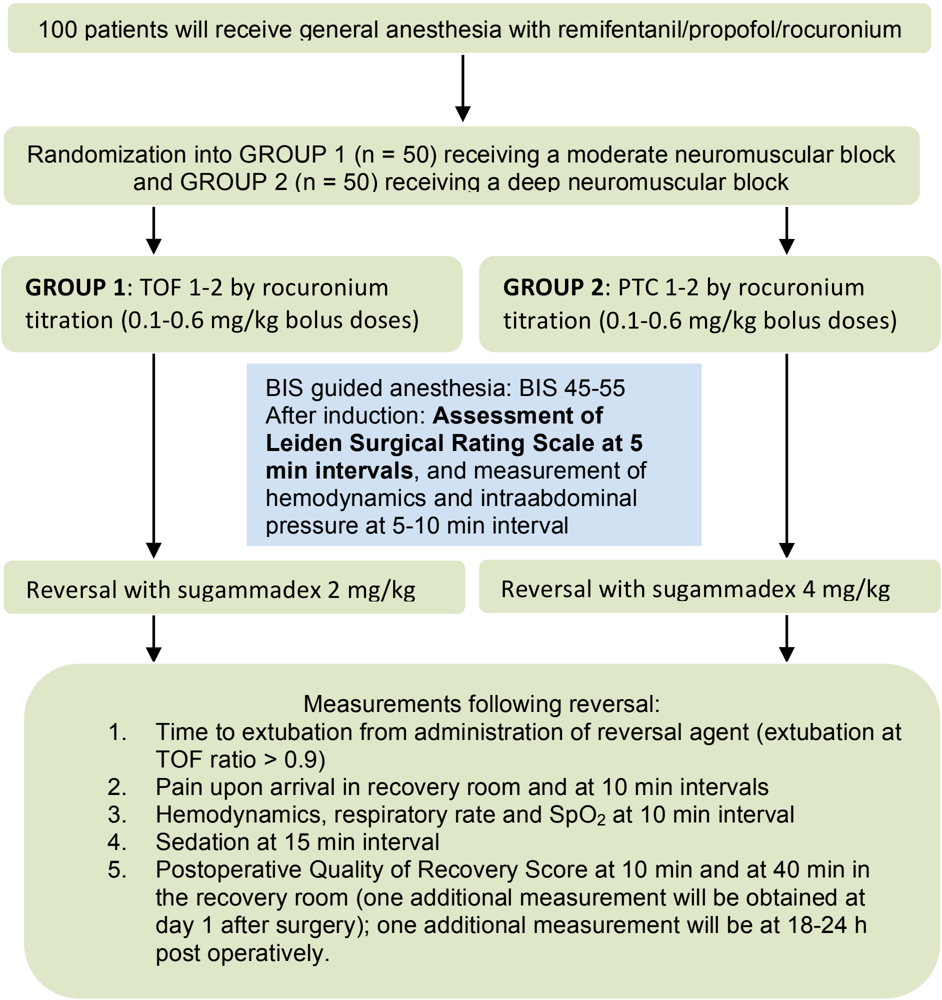

4. Study procedures

This is a randomized controlled parallel study on the effect of deep *versus* moderate rocuronium-induced NMB (PTC 1-2 *vs.* TOF 1-2) in patients with morbid obesity undergoing elective bariatric laparoscopic surgery under propofol/remifentanil anesthesia.

100 patients (18-65 yrs) that will undergo elective bariatric surgery under general anesthesia will be randomized to a moderate neuromuscular block according to standard of practice with TOF values of 1 or 2 (GROUP 1) or a deep neuromuscular block with TOF values of zero and a post-tetanic count of 1 or 2 (GROUP 2). Anesthesia will be induced with propofol and rocuronium (in GROUP 1: dose = 0.6 mg/kg, in group 2: dose = 1 mg/kg) and maintained with propofol and remifentanil according to the fast track protocol for bariatric surgery. The TOF and PTC will be measured at 5 min interval; When the TOF and PTC are off target additional rocuronium will be administered by bolus administrations of 0.1-0.2 mg/kg or a continuous infusion of 20-40 mg per h. **Dosing is based on ideal body weight (men = length in cm – 100; women = length on cm – 105).** During surgery, the target end-tidal PCO_2 will be between 4.5 and 5.5 kPa.

During surgery the surgeon will be asked to score the quality of the operating field using the 5-point Leiden Surgical Rating Scale at 10 min intervals. Additionally the following variables will be collected at 10 min interval on the Case Record Form (CRF): blood pressure, heart rate, BIS, intra-abdominal pressure, end-tidal PCO_2 and pulmonary pressure. Additionally 30 s video snippets and/or 1 or two photos will be taken during the time of scoring.

At the end of surgery all anesthetic administration will be stopped. In GROUP 1 sugammadex will be administered in a dose of 2 mg/kg, in GROUP 2 in a dose of 4 mg/kg. The time to a TOF ratio > 0.9 will be collected on the CRF as well as well as all doses of the drugs used including rocuronium, remifentanil, propofol and sugammadex. After extubation the patient will be transported to the postanesthesia care unit.

In the PACU the following data will be collected: Pain (using an 11 point rating scale), sedation (using an 11 point rating scale), SpO_2, blood pressure, heart rate, breathing rate and PQRS. All drugs given to the patient will be collected on the CRF. Additional PQRS will be obtained every 6-hours postoperatively. On the ward pain will be scored twice (8 PM day of surgery and 2 PM next day). We will discriminate between superficial wound pain, deep tissue pain and referred shoulder pain.

The study will be registered at clinicaltrials.gov (or a similar register such as www.trialregister.nl).

5. Power and Statistical Analysis

The investigator will be responsible for the data analysis. The analysis will be performed on a blinded data set after medical/scientific review has been completed and all protocol violations have been identified and the data set has been declared complete.

There are two primary end-points: The Leiden Surgical rating Scale (see table above) and postoperative pain. The hypothesis of the study is that a deep neuromuscular block will coincide with improved surgical conditions as compared to a standard of care block and less postoperative pain.

Secondary end-points include: hemodynamics during surgery (blood pressure, heart rate); and sedation/SpO_2/blood pressure/heart rate in the recovery period. The hypotheses are: Deep block coincides with optimal hemodynamic conditions during surgery; Deep block coincides with less pain in the recovery room albeit without any cardiorespiratory compromise.

Sample size: Powering the study on Surgical Rating Scale: The estimated mean difference between the treatment groups is conservatively estimated to be at 0.5 (but probably > 1), and combined with various assumptions on the variability (that is anticipated to be approximately 0.4), a sample size of 20 per group would provide at least 90% power to observe the expected difference (see table below):

MEAN DIFFERENCE between treatments

	SD 0.4	SD 0.45	SD 0.5
0.4	23	28	34
0.45	**18**	23	27
0.5	15	**19**	23
0.55	13	16	**19**
0.6	11	13	16

Table: Sample size per group by range of mean group differences and standard deviations (SD) at 90% power.

Powering postoperative pain: Assuming a difference in pain score between the two treatment groups of 1.0 (numerical ratings scale), a SD of 1.5 (CV 150%), and alpha of 0.05, the study has a power > 0.9 to detect a difference with 100 subjects. We therefore decided to enrol 100 patients (50 per group) in this study allowing the detection of a difference in Surgical rating Scale and pain in the recovery room. This approach ensures adequate power to detect significant and clinically relevant differences in the surgical rating scale and postoperative pain. We expect no difference in PQRS, sedation and respiratory parameters.

Statistical analysis will by analysis of variance or t-test as specified by Martini et al. Br J Anaesth 2014. Descriptive and inferential statistics were used for analysis. All data are first tested for normality by a Q-Q plot, a Kolmogorov-Smirnov and a Levene's test.

Descriptive statistics are used to outline characteristics of included patients. Continuous normally distributed variables are expressed by their mean and standard deviation, not normally distributed data by their median and range. To test groups, categorical variables are tested using the Pearson's Chi-square test or Fisher's exact test, when appropriate. Normally distributed continuous data are tested with Students t-test and in case of skewed data, with the Mann-Whitney U test.

Amendment: After consultation with a statistician we decided to analyze the Surgical Rating Scale and the pain score as continuous variables, as we are dealing with at least 5 categorical scales (see Martini et al., 2014). Statistical analysis on the Surgical Rating Scale and pain score in the post anesthesia care unit will then be by linear-mixed model with an autoregressive covariance structure. *Post hoc* analysis was by t-test (with $p < 0.01$ considered significant). Pain scores on the ward (wound pain, deep pain shoulder pain) will be averaged and the group comparison will be by t-test.

Amendment: Comparison of the Leiden Surgical Rating Scale among surgeons

In order to get an indication of the validity of the Leiden Surgical Rating Scale among surgeons we will add a third post hoc group of 50 patient that will receive standard of care, ie. just one 30 mg rocuronium infusion dose at the beginning surgery. This will lead to three NMB groups (single dose, moderate NMB and deep NMB). For each of the surgeons that will participate in the study (currently it is expected that three surgeon will participate) we will create a dose-response relationship of dose (the level of NMB) vs surgical rating scale. We will assess 1) whether dose dependency is observed for the surgeons and 2) whether the variability (with-in observer variability) in the data is similar among the surgeons.

7. References

Martini, Boon, Bevers, Aarts, Dahan. Evaluation of surgical conditions during laparoscopic surgery in patients with moderate vs deep neuromuscular block. Br J Anaesth. 2014; 112: 498-505.

Boon, Martini, Aarts, Bevers, Dahan. Effect of variations in depth of neuromuscular blockade on rating of surgical conditions by surgeon and anesthesiologist in patients undergoing laparoscopic renal or prostatic surgery (BLISS trial): study protocol for a randomized controlled trial. Trials. 2013 Mar 1; 14: 63

Dubois, Putz, Jamart, Marotta, Gourdin, Donnez. Deep neuromuscular block improves surgical conditions during laparoscopic hysterectomy: a randomised controlled trial. Eur J Anaesthesiol. 2014; 31: 430-6.

Carron, Gasparetto, Vidigni, Foletto. Laparoscopic surgery in a morbidly obese, high-risk cardiac patient: the benefits of deep neuromuscular block and sugammadex. Br J Anaesth. 2014 Jul;113(1):186-7.

Carron, Veronese, Foletto, Ori. Sugammadex allows fast-track bariatric surgery. Obes Surg. 2013; 23: 1558-63.

Amorim, Lagarto, Gomes, Esteves, Bismarck, Rodrigues, Nogueira. Neostigmine vs. sugammadex: observational cohort study comparing the quality of recovery using the postoperative quality recovery scale. Acta Anaesthesiol Scand 2014; 58: 1101-1110.
